# Exploratory Analysis of Systemic Inflammatory Indices Across Locoregional Tumor Progression Parameters in Cervical Cancer: The CERTIFIKO Prospective Study

**DOI:** 10.3390/medicina62081570

**Published:** 2026-08-17

**Authors:** Carlo Ronsini, Antonino Di Nuzzo, Maria Cristina Solazzo, Cono Scaffa, Costanza Ascione, Vito Chiantera

**Affiliations:** 1 Unit of Gynecologic Oncology, National Cancer Institute, IRCCS, Fondazione “G. Pascale”, 80131 Naples, Italy; dinuzzo.antonino@gmail.com (A.D.N.); mariacristinasolazzo@gmail.com (M.C.S.); c.scaffa@istitutotumori.na.it (C.S.); costanzaascione@gmail.com (C.A.); vito.chiantera@istitutotumori.na.it (V.C.); 2 Unit of Gynecology and Obstetrics, Policlinico “G. Martino”, Department of Human Pathology of Adult and Childhood “G. Barresi”, University of Messina, 98100 Messina, Italy; 3 Unit of Gynecology and Obstetrics, Department of Woman, Child and General and Specialized Surgery, University of Campania “Luigi Vanvitelli”, 80138 Naples, Italy

**Keywords:** cervical cancer, systemic inflammatory indices, tumor progression, lymphovascular space invasion, parametrial involvement

## Abstract

*Background and Objectives:* Systemic inflammatory indices derived from routine blood tests have been proposed as potential biomarkers reflecting tumor behavior. This study aimed to evaluate their association with parameters of locoregional tumor progression in cervical cancer. *Materials and Methods:* This prospective observational study (CERTIFIKO) included 79 patients with cervical cancer enrolled between July 2023 and December 2025. This study was registered on ClinicalTrials.gov (NCT05673252). Pre-treatment blood samples were used to calculate the neutrophil-to-lymphocyte ratio (NLR), monocyte-to-lymphocyte ratio (MLR), platelet-to-lymphocyte ratio (PLR), systemic inflammatory response (SIR), and systemic inflammatory response index (SIRI). Tumor progression parameters included stromal infiltration, parametrial involvement, lymphovascular space invasion (LVSI), fornix involvement, lymph node status, histotype, and grading. Associations were assessed using non-parametric tests and linear regression models. *Results:* Stromal infiltration was significantly associated with PLR (*p* = 0.011), MLR (*p* = 0.010), and NLR (*p* = 0.010). Parametrial involvement was associated with MLR (*p* = 0.004) and NLR (*p* = 0.012). Diffuse LVSI was associated with PLR (*p* = 0.002), NLR (*p* = 0.006), SIR (*p* = 0.008), and SIRI (*p* = 0.034). In multivariable linear regression models, diffuse LVSI remained associated with higher PLR and NLR values, whereas parametrial involvement remained associated with higher MLR, NLR, and SIRI. No significant associations were observed for fornix involvement, histotype, or lymph node status. *Conclusions:* Systemic inflammatory indices were associated with parameters of locoregional tumor progression in this exploratory cohort of patients with cervical cancer. These findings warrant further evaluation in larger studies before any potential clinical application.

## 1. Introduction

Cervical cancer is characterized by a pattern of local tumor dissemination, in which the progressive involvement of anatomical structures adjacent to the cervix, such as the parametria, vaginal fornices, and lymphovascular spaces, plays a central role in disease staging and directly influences therapeutic decision-making [1,2]. The identification of these features is therefore crucial in defining the most appropriate treatment strategy, including the choice between primary surgery and chemoradiation [3]. However, the assessment of local tumor extension remains partly presumptive, and in some cases, the true extent of disease is only accurately identified after surgical treatment and histopathological examination [3,4]. Current staging systems are primarily based on clinical evaluation and supported by imaging techniques such as magnetic resonance imaging (MRI) and positron emission tomography (PET), which have become essential tools for assessing locoregional spread and nodal involvement [5]. Nevertheless, these modalities are associated with high costs and are not uniformly available worldwide, potentially limiting their accessibility in certain healthcare settings [6]. In parallel, a growing body of evidence has demonstrated that tumor progression in several solid malignancies is accompanied by measurable changes in the systemic inflammatory response [7,8]. Alterations in circulating immune cell populations, particularly neutrophils, lymphocytes, monocytes, and platelets, can be detected through routine blood tests and have been associated with tumor burden and disease progression [9,10]. These changes may reflect the interaction between tumor cells and the host immune system, even in early phases of tumor dissemination [11]. In this context, inflammatory indices derived from complete blood count represent simple, low-cost, and widely available biomarkers that could potentially provide additional information on tumor behavior [12,13].

### Objective

Within this framework, the objective of the present study was to evaluate whether systemic inflammatory indices, including the neutrophil-to-lymphocyte ratio (NLR) [14], monocyte-to-lymphocyte ratio (MLR) [15], platelet-to-lymphocyte ratio (PLR) [16], systemic inflammatory response index (SIR) [10,11,12,13], and systemic inflammatory response index of inflammation (SIRI) [7,13,14,15,16], obtainable from routine blood tests, are associated with parameters of locoregional tumor spread in cervical cancer, with the goal of identifying measurable indicators of tumor progression that could complement existing diagnostic methods [7,13,14,15,16]. However, most available studies have focused on dichotomized biomarkers or survival outcomes, often relying on predefined cut-off values that may limit the interpretability of continuous biological variation. In addition, the relationship between systemic inflammatory indices and specific local parameters of tumor progression remains incompletely characterized. The present study aimed to address these gaps by evaluating multiple inflammatory indices as continuous biological outcomes in relation to documented parameters of locoregional tumor progression, including stromal infiltration, parametrial involvement, and lymphovascular space invasion. Therefore, the primary analytical question was not whether inflammatory indices predict the presence of a pathological feature, but whether different states of local tumor progression are accompanied by measurable variations in systemic inflammatory status. Within this exploratory framework, inflammatory indices were retained as continuous dependent variables in order to quantify differences in their values across categories of documented tumor progression.

## 2. Materials and Methods

### 2.1. Study Design

A prospective observational study was conducted to evaluate the association between histopathological markers of tumor aggressiveness and systemic inflammatory indices in patients with cervical cancer. The study was designed a priori and reported in accordance with the Strengthening the Reporting of Observational Studies in Epidemiology (STROBE) guidelines [17,18,19].

### 2.2. Settings

This study was conducted through an integrated academic-oncology pathway involving IRCCS Istituto Nazionale Tumori “G. Pascale” and the University of Campania “Luigi Vanvitelli”. The two institutions operate within the same university-associated clinical network; laboratory procedures were standardized across the pathway, and histopathological assessment is centralized. Because recruitment and laboratory processing were managed as a shared pathway rather than as two analytically independent center strata, the study database did not encode a separate center variable, and a center-stratified analysis was not performed. Patients were consecutively enrolled between July 2023 and December 2025. This study protocol received approval from the Ethics Committee of the University of Campania “Luigi Vanvitelli” (protocol 0035558/i, 24 November 2022), was registered on ClinicalTrials.gov (NCT05673252), and all participants provided written informed consent.

### 2.3. Study Population

**Figure 1:**
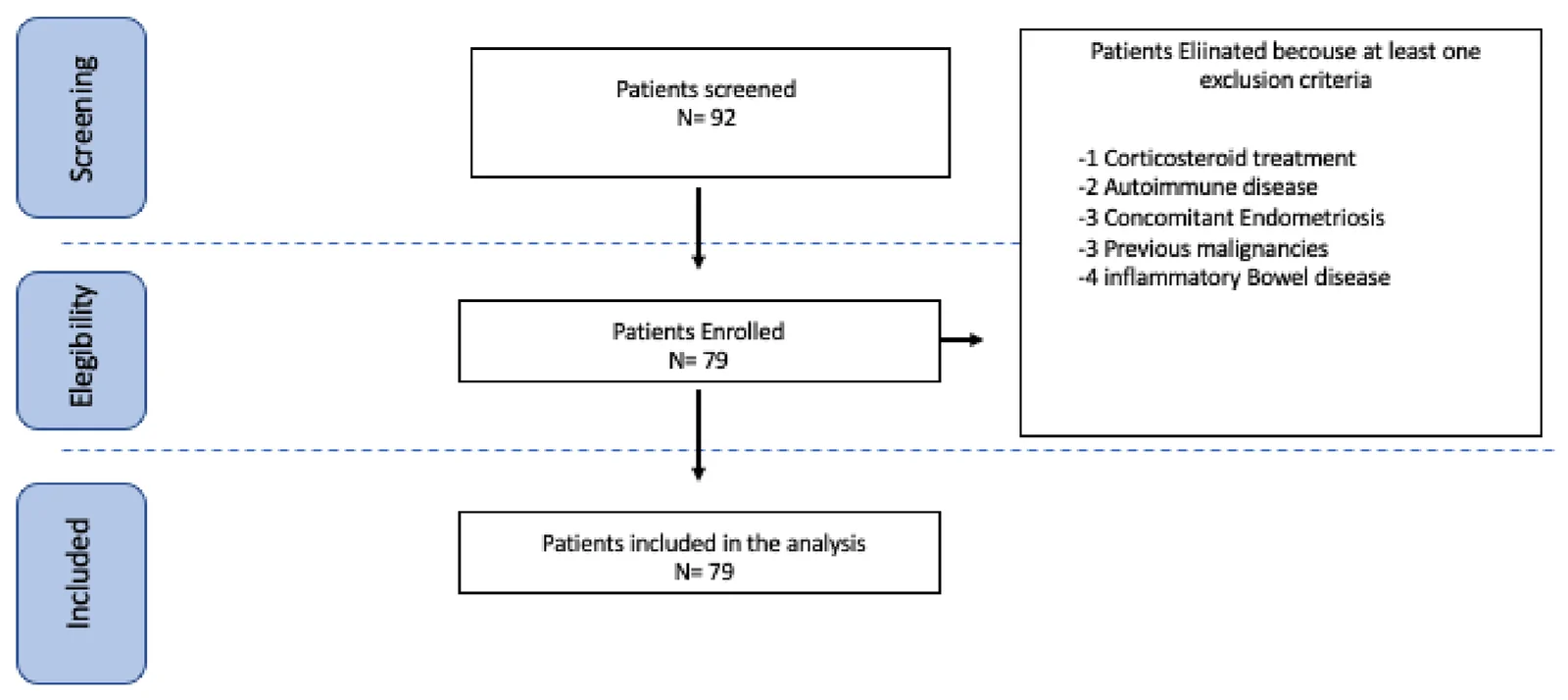
Enrollment flow chart.

Patients were eligible if they were aged ≥ 18 years, had a histological or radiological diagnosis of cervical cancer, had complete clinical, laboratory, and tumor-assessment data, and had a pre-treatment blood sample collected within seven days before treatment. Among 92 screened patients, 13 were excluded: corticosteroid therapy (*n* = 2), previous malignancy (*n* = 2; bladder and breast cancer), inflammatory bowel disease (*n* = 3), autoimmune disease (*n* = 4), and a history of endometriosis (*n* = 3). One patient met both the inflammatory bowel disease and autoimmune disease criteria; therefore, exclusion reasons total fourteen, although thirteen unique patients were excluded. No patient was excluded solely because of missing laboratory, imaging, or pathological data (Figure 1).

### 2.4. Variables

Clinical and demographic variables collected at baseline included age, body mass index, menopausal status, prior abdominal surgery, comorbidities, treatment type, FIGO stage, and maximum tumor diameter. Tumor progression variables included stromal infiltration, vaginal-fornix involvement, lymphovascular space invasion (LVSI), parametrial involvement, lymph node status, histotype, and grade. For 23 patients whose initial treatment-planning assessment relied on imaging, subsequent conization provided histopathological confirmation of diagnosis, stromal infiltration, LVSI, histotype, grade, and other pathological variables. Parametrial involvement remained a radiological variable in these cases and was independently assessed by two radiologists blinded to laboratory data. This mixed-source approach was retained because it reflects the real-world pre-treatment pathway; its potential for classification heterogeneity is acknowledged as a limitation. All histopathological assessments were centrally reviewed by two pathologists blinded to clinical and laboratory findings [20,21]. Missing values were retained and are reported with variable-specific denominators in the descriptive and regression tables.

### 2.5. Laboratory Procedures

Peripheral venous blood samples were collected within seven days before treatment and before biopsy, conization, surgery, radiotherapy, chemotherapy, or any other diagnostic or therapeutic invasive procedure that could modify systemic inflammatory indices. Samples were processed according to standardized procedures within the shared academic-oncology laboratory network [22,23,24]. Automated hematology analyzers underwent routine internal and external quality-control and calibration procedures, and results were expressed as absolute cell counts per microliter.

### 2.6. Statistical Analysis

Categorical variables were summarized as counts and percentages using the available denominator, and continuous variables as medians and interquartile ranges. Wilcoxon rank-sum or Kruskal–Wallis tests were used for unadjusted group comparisons. The a priori sample-size calculation (Cohen’s f^2^ = 0.15, alpha = 0.05, power = 80%, three numerator degrees of freedom) yielded 77 patients and supported only the prespecified exploratory framework; it did not establish adequate power for every final model, index, category-specific contrast, or sensitivity analysis. Separate linear regression models were fitted for NLR, MLR, PLR, SIR, and SIRI as continuous dependent variables. The same predictors were included in each primary multivariable model: stromal infiltration, parametrial involvement, and LVSI, with superficial infiltration, no parametrial involvement, and no/focal LVSI as reference categories. Model results include N, beta coefficients, 95% confidence intervals, adjusted R^2^, and the overall model *p*-value. Residual distribution was assessed using residual plots and the Shapiro–Wilk test; homoscedasticity using residual-versus-fitted plots and the Breusch–Pagan test; and influential observations using Cook’s distance. Because the primary predictors were categorical, a continuous-predictor linearity assumption was not applicable to those terms; linearity of age was inspected in age-adjusted sensitivity models. Residual non-normality and several potentially influential observations were identified, while heteroscedasticity was most evident for PLR. Therefore, sensitivity analyses used HC3 heteroscedasticity-consistent standard errors and log1p-transformed inflammatory indices. The direction of the principal estimates was materially unchanged. Age-adjusted models were additionally fitted. Given multiple correlated analyses, unadjusted *p*-values remain the primary exploratory results; a supplementary Holm-adjusted analysis across the 30 group comparisons was added. No association remained below 0.05 after Holm adjustment, reinforcing the hypothesis-generating interpretation. Analyses were performed in R and independently reproduced for revision.

### 2.7. Risk of Bias

The models were intentionally parsimonious and minimally adjusted. The same three tumor progression predictors were used for each inflammatory-index outcome, while age was examined separately in sensitivity analyses. BMI could not be included reliably because 21 values were missing; additional adjustment for comorbidity burden, menopausal status, tumor size, FIGO stage, and center would have produced unstable models in this cohort. Residual confounding therefore remains possible. The use of radiological parametrial assessment in 23 patients was retained to reproduce routine clinical practice; because these patients subsequently received conization confirming all pathological variables except parametrial involvement, exclusion of the entire subgroup would discard valid pathological information and alter the target clinical population.

## 3. Results

### 3.1. Characteristics

A total of 92 patients were screened, and 79 were included. Thirteen unique patients were excluded: corticosteroid therapy (*n* = 2), previous malignancy (*n* = 2), inflammatory bowel disease (*n* = 3), autoimmune disease (*n* = 4), and endometriosis (*n* = 3); one patient fulfilled both inflammatory bowel disease and autoimmune disease criteria. No exclusion was due solely to missing laboratory, imaging, or pathological data. Variable-specific missingness is reported in Table 1.

### 3.2. Outcomes

**Figure 2:**
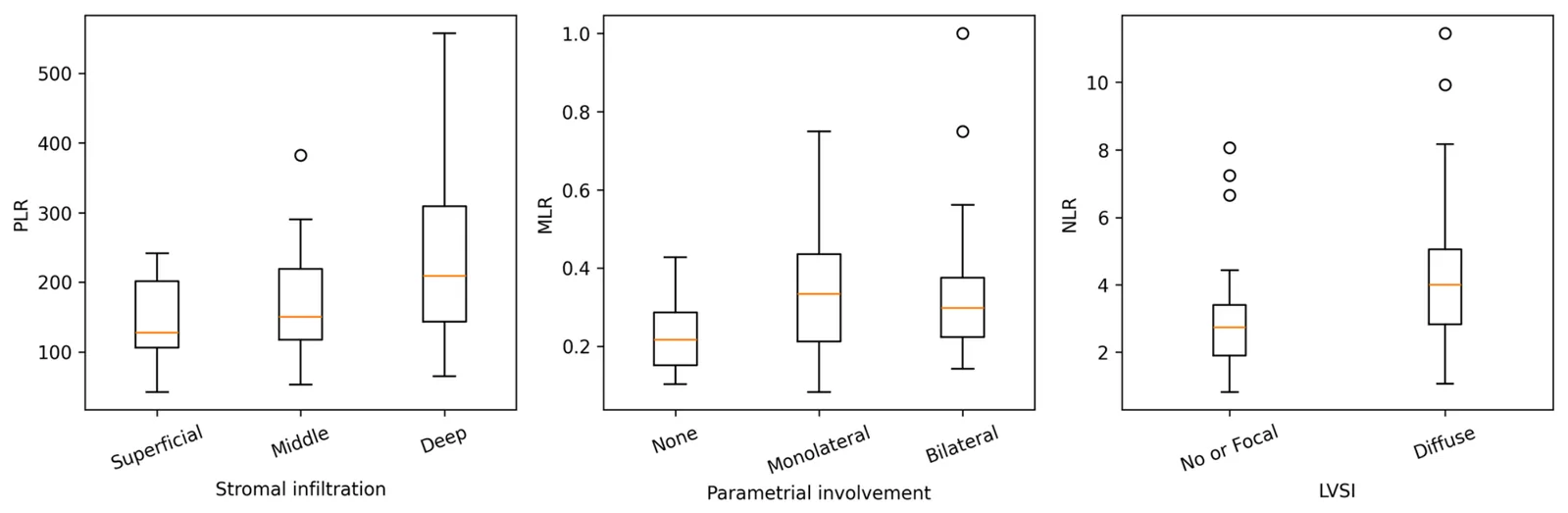
Simplified presentation of representative associations between systemic inflammatory indices and locoregional tumor progression parameters: PLR across stromal-infiltration categories, MLR across parametrial-involvement categories, and NLR according to LVSI. Complete plots for all indices and parameters are provided in the Supplementary Material section.

Inflammatory indices were compared across the prespecified tumor progression parameters. To improve readability, the main Figure 2 displays only three representative associations central to the study: PLR across stromal-infiltration categories, MLR across parametrial-involvement categories, and NLR according to LVSI. The complete set of plots and extended numerical comparisons has been moved to the Supplementary Materials.

Stromal infiltration showed statistically significant associations with PLR (128 vs. 150 vs. 209; *p* = 0.011), MLR (0.22 vs. 0.19 vs. 0.29; *p* = 0.010), and NLR (2.58 vs. 3.26 vs. 4.06; *p* = 0.010). No significant associations were observed for SIR (581 vs. 865 vs. 918; *p* = 0.13) and SIRI (64 vs. 48 vs. 73; *p* = 0.087). Parametrial involvement was associated with MLR (0.22 vs. 0.33 vs. 0.30; *p* = 0.004) and NLR (2.90 vs. 4.42 vs. 3.93; *p* = 0.012), but no significant relationships were found for PLR, SIR, and SIRI. Diffuse LVSI was significantly associated with PLR (135 vs. 198; *p* = 0.002), NLR (2.73 vs. 4.00; *p* = 0.006), SIR (609 vs. 1024; *p* = 0.008), and SIRI (54 vs. 73; *p* = 0.034), although MLR did not reach significance (0.21 vs. 0.27; *p* = 0.095). No significant links were identified between inflammatory indices and involvement of the fornices, histotype, or lymph node status.

These results are summarized in Table 2.

### 3.3. Primary Exploratory Analysis: Inflammatory Indices as Continuous Dependent Variables

In accordance with the primary exploratory objective of the study, each inflammatory index was modeled as a continuous dependent variable, while parameters of documented locoregional tumor progression were entered as categorical explanatory variables. Therefore, the reported beta coefficients quantify absolute differences in inflammatory index values across tumor progression categories and should not be interpreted as estimates of the probability of pathological progression.

In univariable linear regression analyses, several parameters of documented locoregional tumor progression were associated with higher values of systemic inflammatory indices. Compared with superficial stromal infiltration, deep stromal infiltration was associated with higher PLR values (β = 85; 95% CI 31–138; *p* = 0.002), higher MLR values (β = 0.09; 95% CI 0.01–0.18; *p* = 0.033), higher NLR values (β = 1.5; 95% CI 0.48–2.6; *p* = 0.005), and higher SIR values (β = 640; 95% CI 19–1261; *p* = 0.043). Compared with the absence of parametrial involvement, monolateral involvement was associated with higher MLR values (β = 0.12; 95% CI 0.03–0.20; *p* = 0.006) and higher NLR values (β = 1.5; 95% CI 0.45–2.6; *p* = 0.006), whereas bilateral involvement was associated with higher PLR values (β = 57; 95% CI 1.9–112; *p* = 0.043), MLR values (β = 0.13; 95% CI 0.05–0.21; *p* = 0.003), NLR values (β = 1.3; 95% CI 0.25–2.3; *p* = 0.016), SIR values (β = 648; 95% CI 34–1261; *p* = 0.039), and SIRI values (β = 49; 95% CI 13–86; *p* = 0.009). Finally, diffuse LVSI, compared with no or focal LVSI, was associated with higher PLR values (β = 71; 95% CI 24–119; *p* = 0.004) and higher NLR values (β = 1.1; 95% CI 0.18–2.1; *p* = 0.020). The relatively large coefficients observed for SIR reflect the larger absolute numerical scale of this composite index and represent differences in SIR units rather than estimates of tumor progression risk. These results are summarized in Table 3.

In multivariable linear regression analyses, using inflammatory indices as continuous dependent variables, diffuse LVSI remained associated with higher PLR values compared with no or focal LVSI (β = 62; 95% CI 9.7–113; *p* = 0.021) and with higher NLR values (β = 1.1; 95% CI 0.05–2.1; *p* = 0.039). Compared with the absence of parametrial involvement, monolateral parametrial involvement remained associated with higher MLR values (β = 0.12; 95% CI 0.03–0.20; *p* = 0.010) and higher NLR values (β = 1.6; 95% CI 0.44–2.7; *p* = 0.007). Bilateral parametrial involvement remained associated with higher MLR values (β = 0.11; 95% CI 0.03–0.20; *p* = 0.010), higher NLR values (β = 1.2; 95% CI 0.10–2.2; *p* = 0.032), and higher SIRI values (β = 45; 95% CI 6.0–83; *p* = 0.024). No statistically significant associations were retained for SIR in the multivariable models. These findings are reported in Table 4.

Sensitivity analyses were performed using age-adjusted models, HC3 heteroscedasticity-consistent standard errors, and log1p-transformed inflammatory indices. The direction of the principal associations was materially unchanged, although residual non-normality and influential observations confirmed that estimates should be interpreted cautiously. Model-level diagnostics and statistics are reported in Supplementary Table S1, complete age-adjusted results in Supplementary Table S2, and Holm-adjusted exploratory comparisons in Supplementary Table S3. None of the 30 group-comparison *p*-values remained below 0.05 after Holm adjustment.

## 4. Discussion

### 4.1. Interpretation of the Results

This prospective study identified cohort-level associations between selected systemic inflammatory indices and specific components of locoregional cervical cancer extension. These findings quantify differences in inflammatory-index values across documented tumor progression categories; they do not establish causality, diagnostic accuracy, or individual-level predictive utility. The loss of formal significance after Holm adjustment further supports interpretation as hypothesis-generating evidence.

### 4.2. Clinical Interpretation

The present findings should not be used to replace or supplement imaging or pathological assessment in clinical decision-making. This study did not evaluate discrimination, calibration, clinically applicable thresholds, incremental value beyond standard assessment, or external validation. Any possible role for inflammatory indices in future resource-limited or multimodal assessment pathways remains a research hypothesis requiring dedicated diagnostic-performance studies [25,26].

### 4.3. Comparison with Existing Literature

The link between systemic inflammatory response and tumor progression aligns with previous research on various solid tumors [14,18,26,27]. Inflammation has long been recognized as a crucial element of cancer biology, affecting tumor growth, invasion, and metastatic ability [28]. The results of this study are consistent with the existing literature and extend current knowledge by providing a more detailed and continuous assessment of inflammatory dynamics in relation to local tumor progression in cervical cancer [7,8,9]. Importantly, our team has previously shown similar associations in endometrial cancer, where inflammatory indices related to factors such as myometrial invasion and LVSI [11,12,13], as well as in adnexal masses [10,11,12], reinforcing the idea that systemic inflammatory response reflects underlying tumor biology. These current findings further support the notion that inflammation-related biomarkers may serve as a common pathway across different gynecologic cancers [8,9,10]. In the present study, inflammatory indices were analyzed as continuous response variables, while documented parameters of tumor progression were entered as explanatory variables. Accordingly, the observed associations describe estimated differences in inflammatory index values across categories of locoregional tumor progression within the present cohort. This analytical framework is consistent with the exploratory objective of the study and does not provide evidence for individual-level clinical stratification or treatment decision-making.

### 4.4. Strengths and Limitations

Strengths include the prospective design, pre-procedure blood sampling, standardized laboratory pathway, centralized pathology review, blinded assessment, and explicit reporting of effect estimates. Important limitations include the modest sample size; multiple correlated analyses; non-normal residuals and influential observations; lack of significance after Holm adjustment; 21 missing BMI values; and inability to adjust comprehensively for comorbidities, menopausal status, tumor size, FIGO stage, or center. Although the 23 initially imaging-assessed patients subsequently underwent conization confirming the pathological variables, parametrial involvement remained radiologically inferred in this subgroup. This reflects real-world practice but may introduce classification heterogeneity. The sample-size calculation supported the general prespecified framework rather than every final analysis, and the multivariable models should be considered exploratory and minimally adjusted.

## 5. Conclusions

In this prospective exploratory cohort, selected systemic inflammatory indices varied across categories of stromal infiltration, parametrial involvement, and LVSI. These estimates describe associations within the present cohort only. They do not establish clinical utility, incremental diagnostic value, or a basis for individual treatment decisions. The absence of associations surviving Holm correction and the limited adjustment for potential confounders require confirmation in larger, independently validated cohorts.

## Figures and Tables

**Table 1:** Baseline clinical, demographic, and tumor progression characteristics of the study population.

Characteristic	*n* = 79 ^1^
Age	56 (45–65)
BMI	25.0 (23.0–29.0)
Missing	21
Menopause	52 (66%)
Previous Abdominal Surgery	41 (52%)
Comorbidity	36 (46%)
Missing	1
Treatment	
Chemoradiation	49 (62%)
LPT	18 (23%)
MIS	12 (15%)
Histotype	
Adenocarcinoma	8 (10%)
Adeno-Squamous	3 (3.8%)
Squamous	68 (86%)
Grading	
1	7 (8.9%)
2	21 (27%)
3	51 (65%)
Stromal Infiltration	
Superficial	20 (25%)
Middle	16 (20%)
Deep	43 (54%)
Fornices	
Negative	45 (57%)
Positive	34 (43%)
LVSI	
No or Focal	26 (33%)
Diffuse	53 (67%)
Parametrium	
None	38 (48%)
Monolateral	19 (24%)
Bilateral	22 (28%)
Lymph nodes	
Negative	37 (47%)
Positive	42 (53%)

^1^ Median (25–75%); *n* (%), LPT Laparotomic, MIS Mini-Invasive Surgery, LVSI Lymphovascular Space Invasion.

**Table 2:** Distribution of systemic inflammatory indices according to tumor progression parameters.

**A. Parametrial involvement**				
Index	None, *n* = 38	Monolateral, *n* = 19	Bilateral, *n* = 22	*p*-value
PLR	143 (108–238)	193 (158–244)	196 (138–280)	0.13
MLR	0.22 (0.15–0.29)	0.33 (0.21–0.44)	0.30 (0.22–0.37)	0.004
NLR	2.90 (1.94–3.88)	4.42 (3.05–5.93)	3.93 (2.70–5.91)	0.012
SIR	822 (432–1281)	1023 (566–1751)	787 (574–1349)	0.3
SIRI	57 (36–93)	83 (63–113)	71 (56–94)	0.094
**B. Vaginal fornix involvement**				
Index	Negative, *n* = 45	Positive, *n* = 34		*p*-value
PLR	163 (118–240)	184 (135–291)		0.5
MLR	0.24 (0.18–0.31)	0.29 (0.21–0.43)		0.052
NLR	3.26 (2.31–4.27)	3.93 (2.70–6.02)		0.14
SIR	819 (507–1330)	924 (500–1553)		0.4
SIRI	64 (38–84)	78 (56–114)		0.081
**C. Stromal infiltration**				
Index	Superficial, *n* = 20	Middle, *n* = 16	Deep, *n* = 43	*p*-value
PLR	128 (106–201)	150 (117–219)	209 (143–310)	0.011
MLR	0.22 (0.18–0.30)	0.19 (0.13–0.29)	0.29 (0.22–0.39)	0.010
NLR	2.58 (1.87–3.54)	3.26 (1.89–5.21)	4.06 (2.81–5.12)	0.010
SIR	581 (475–1123)	865 (459–1624)	918 (579–1524)	0.13
SIRI	64 (46–81)	48 (34–89)	73 (52–108)	0.087
**D. Histotype**				
Index	Adeno-Squamous, *n* = 3	Adenocarcinoma, *n* = 8	Squamous, *n* = 68	*p*-value
PLR	208 (158–278)	130 (110–180)	171 (131–243)	0.4
MLR	0.25 (0.23–0.35)	0.22 (0.17–0.25)	0.26 (0.20–0.36)	0.3
NLR	3.47 (3.22–4.79)	2.56 (1.45–3.47)	3.61 (2.31–4.79)	0.13
SIR	1376 (1066–1641)	552 (432–1172)	833 (511–1339)	0.3
SIRI	83 (74–111)	52 (40–96)	66 (42–97)	0.4
**E. Lymphovascular space invasion**				
Index	No or Focal, *n* = 26	Diffuse, *n* = 53		*p*-value
PLR	135 (104–190)	198 (142–291)		0.002
MLR	0.21 (0.15–0.31)	0.27 (0.21–0.36)		0.095
NLR	2.73 (1.90–3.39)	4.00 (2.82–5.07)		0.006
SIR	609 (464–824)	1023 (561–1595)		0.008
SIRI	54 (40–82)	72 (43–110)		0.034
**F. Lymph node status**				
Index	Negative, *n* = 37	Positive, *n* = 42		*p*-value
PLR	160 (116–212)	186 (136–287)		0.2
MLR	0.25 (0.20–0.33)	0.23 (0.19–0.33)		0.6
NLR	3.22 (2.32–4.42)	3.69 (2.38–4.67)		0.4
SIR	792 (496–1330)	865 (533–1531)		0.3
SIRI	64 (43–96)	66 (43–97)		0.8

Data are presented as median (Q1–Q3). *p*-values were calculated using the Wilcoxon rank-sum test for two-group comparisons and the Kruskal–Wallis test for three-group comparisons. IQR, interquartile range; LVSI, lymphovascular space invasion; MLR, monocyte-to-lymphocyte ratio; NLR, neutrophil-to-lymphocyte ratio; PLR, platelet-to-lymphocyte ratio; SIR, systemic inflammation response; SIRI, systemic inflammation response index.

**Table 3:** Univariable linear regression models evaluating inflammatory indices as continuous dependent variables.

	NLR			MLR			PLR			SIR			SIRI		
Characteristic	Beta	95% CI	*p*-Value	Beta	95% CI	*p*-Value	Beta	95% CI	*p*-Value	Beta	95% CI	*p*-Value	Beta	95% CI	*p*-Value
Stromal Infiltration															
Superficial	—	—		—	—		—	—		—	—		—	—	
Middle	1.1	−0.25, 2.4	0.11	−0.02	−0.12, 0.09	0.7	33	−34, 99	0.3	420	−350, 1190	0.3	2.3	−44, 49	>0.9
Deep	1.5	0.48, 2.6	0.005	0.09	0.01, 0.18	0.033	85	31, 138	0.002	640	19, 1261	0.043	34	−3.5, 72	0.074
Parametrial Involvement															
None	—	—		—	—		—	—		—	—		—	—	
Monolateral	1.5	0.45, 2.6	0.006	0.12	0.03, 0.20	0.006	38	−20, 96	0.2	323	−321, 967	0.3	19	−19, 58	0.3
Bilateral	1.3	0.25, 2.3	0.016	0.13	0.05, 0.21	0.003	57	1.9, 112	0.043	648	34, 1261	0.039	49	13, 86	0.009
LVSI															
No or Focal	—	—		—	—		—	—		—	—		—	—	
Diffuse	1.1	0.18, 2.1	0.020	0.04	−0.04, 0.12	0.3	71	24, 119	0.004	487	−62, 1037	0.081	22	−12, 56	0.2

Beta coefficients represent absolute mean differences in each inflammatory index compared with the indicated reference category. For SIR, coefficients appear numerically larger because of the intrinsic scale of the index, calculated as neutrophils × platelets/lymphocytes. LVSI: lymphovascular space invasion; CI: confidence interval.

**Table 4:** Multivariable linear regression models evaluating inflammatory indices as continuous dependent variables.

	NLR			MLR			PLR			SIR			SIRI		
Characteristic	Beta	95% CI	*p*-Value	Beta	95% CI	*p*-Value	Beta	95% CI	*p*-Value	Beta	95% CI	*p*-Value	Beta	95% CI	*p*-Value
LVSI															
No or Focal	—	—		—	—		—	—		—	—		—	—	
Diffuse	1.1	0.05, 2.1	0.039	0.04	−0.04, 0.12	0.3	62	9.7, 113	0.021	411	−199, 1020	0.2	20	−17, 57	0.3
Parametrial Involvement															
None	—	—		—	—		—	—		—	—		—	—	
Monolateral	1.6	0.44, 2.7	0.007	0.12	0.03, 0.20	0.010	36	−21, 93	0.2	314	−357, 985	0.4	18	−22, 59	0.4
Bilateral	1.2	0.10, 2.2	0.032	0.11	0.03, 0.20	0.010	45	−9.8, 100	0.11	584	−60, 1227	0.075	45	6.0, 83	0.024
Stromal Infiltration															
Superficial	—	—		—	—		—	—		—	—		—	—	
Middle	0.45	−0.89, 1.8	0.5	−0.05	−0.16, 0.05	0.3	2.8	−66, 72	>0.9	202	−608, 1013	0.6	−9.2	−58, 40	0.7
Deep	0.62	−0.56, 1.8	0.3	0.03	−0.06, 0.13	0.5	43	−18, 103	0.2	289	−425, 1004	0.4	13	−30, 56	0.5

Beta coefficients represent absolute mean differences in each inflammatory index compared with the indicated reference category. For SIR, coefficients appear numerically larger because of the intrinsic scale of the index, calculated as neutrophils × platelets/lymphocytes. LVSI: lymphovascular space invasion; CI: confidence interval.

## Data Availability

All data and the methodological process for their calculation have been deposited on Zenodo at https://doi.org/10.5281/zenodo.19322128. Their use is public.

## Supplementary Materials

The following supporting information can be downloaded at: https://www.mdpi.com/article/10.3390/medicina62081570/s1. Figure S1. Distribution of inflammatory indices according to parameters of documented tumor progression; Table S1. Primary multivariable model diagnostics and model-level statistics; Table S2. Age-adjusted sensitivity analyses; Table S3. Holm-adjusted exploratory group comparisons; Table S4. HC3 and log-transformed sensitivity analyses.

## Abbreviations

The following abbreviations are used in this manuscript:

LVSI	Lymphovascular Space Invasion
MLR	Monocyte-to-Lymphocyte Ratio
MRI	Magnetic Resonance Imaging
NLR	Neutrophil-to-Lymphocyte Ratio
PET	Positron Emission Tomography
PLR	Platelet-to-Lymphocyte Ratio
SIR	Systemic Inflammation Response
SIRI	Systemic Inflammation Response Index
