# Supplementary material for: Exploratory Analysis of Systemic Inflammatory Indices Across Locoregional Tumor Progression Parameters in Cervical Cancer: The CERTIFIKO Prospective Study
Source: Medicina (Kaunas). 2026 Aug 17;62(8):1570. doi: 10.3390/medicina62081570 (PMC13515872; doi:10.3390/medicina62081570)

Supplementary Tables

Table S1. Primary multivariable model diagnostics and model-level statistics.

Outcome	N	Adjusted R^2	Overall model p	Shapiro–Wilk p	Breusch–Pagan p	Cook's D > 4/N
NLR	79	0.165	0.003	<0.001	0.534	6
MLR	79	0.152	0.004	<0.001	0.202	5
PLR	79	0.149	0.005	0.019	0.035	4
SIR	79	0.046	0.133	<0.001	0.210	5
SIRI	79	0.066	0.075	<0.001	0.057	3

Table S2. Age-adjusted sensitivity analyses.

Outcome	N	Adjusted R^2	Overall model p	Age β	Age p
NLR	79	0.154	0.006	-0.003	0.850
MLR	79	0.145	0.008	<0.001	0.524
PLR	79	0.149	0.007	-0.881	0.314
SIR	79	0.033	0.210	-1.269	0.902
SIRI	79	0.055	0.119	-0.271	0.663

Table S3. Holm-adjusted exploratory group comparisons.

Tumor progression parameter	Index	N	Unadjusted p	Holm-adjusted p
lvsi	PLR	79	0.002	0.065
parametri_assoluto	MLR	79	0.004	0.112
lvsi	NLR	79	0.006	0.167
lvsi	SIR	79	0.008	0.217
inf_stromale_in_mm	MLR	79	0.010	0.254
inf_stromale_in_mm	NLR	79	0.010	0.254
inf_stromale_in_mm	PLR	79	0.011	0.272
parametri_assoluto	NLR	79	0.012	0.284
lvsi	SIRI	79	0.034	0.742
fornici	MLR	79	0.052	1.000
fornici	SIRI	79	0.081	1.000
inf_stromale_in_mm	SIRI	79	0.087	1.000
parametri_assoluto	SIRI	79	0.094	1.000
lvsi	MLR	79	0.095	1.000
inf_stromale_in_mm	SIR	79	0.127	1.000
parametri_assoluto	PLR	79	0.131	1.000
istotipo	NLR	79	0.134	1.000
fornici	NLR	79	0.143	1.000
linfnodi	PLR	79	0.195	1.000
parametri_assoluto	SIR	79	0.259	1.000
istotipo	SIR	79	0.279	1.000
linfnodi	SIR	79	0.280	1.000
istotipo	MLR	79	0.317	1.000
fornici	SIR	79	0.370	1.000
linfnodi	NLR	79	0.374	1.000
istotipo	PLR	79	0.445	1.000
istotipo	SIRI	79	0.450	1.000
fornici	PLR	79	0.476	1.000
linfnodi	MLR	79	0.579	1.000
linfnodi	SIRI	79	0.757	1.000

Table S4. HC3 and log-transformed sensitivity analyses.

Outcome	N	Primary adjusted R^2	Primary model p	HC3 model interpretation	Log-model adjusted R^2	Log-model p
NLR	79	0.165	0.003	Coefficient directions materially unchanged	0.198	<0.001
MLR	79	0.152	0.004	Coefficient directions materially unchanged	0.169	0.002
PLR	79	0.149	0.005	Coefficient directions materially unchanged	0.163	0.003
SIR	79	0.046	0.133	Coefficient directions materially unchanged	0.083	0.045
SIRI	79	0.066	0.075	Coefficient directions materially unchanged	0.098	0.027

Supplementary Figures

Figure S1. Distribution of inflammatory indices according to parameters of documented tumor progression.

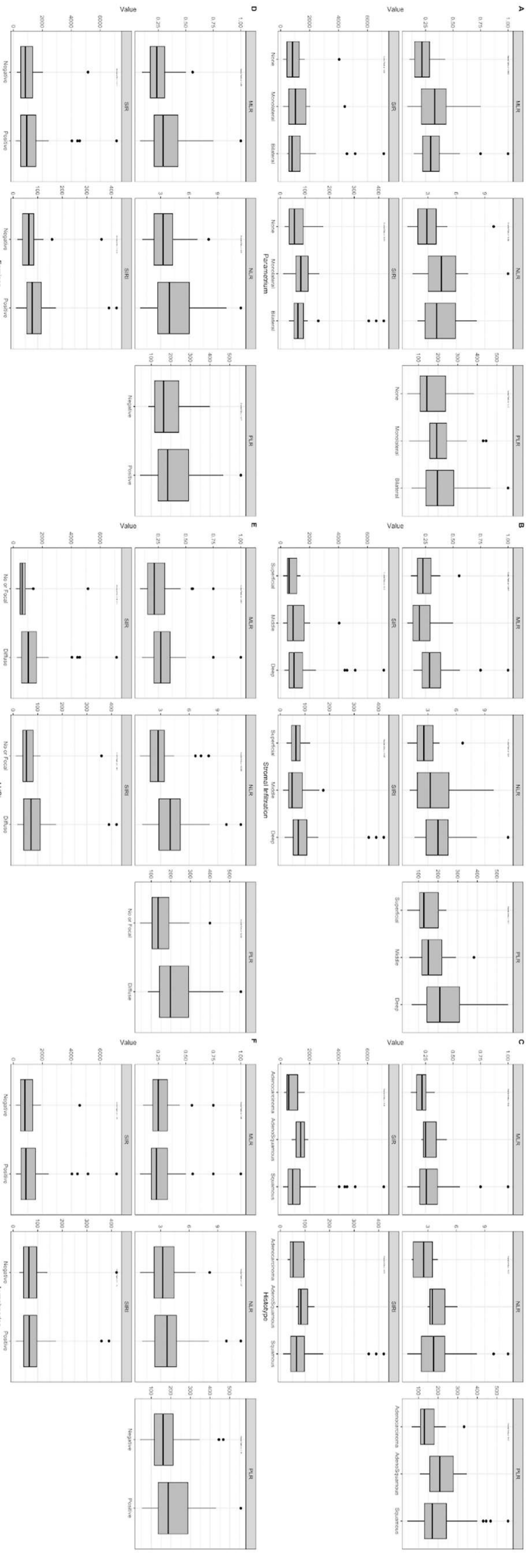

Supplement: Supplementary file 1 [file medicina-62-01570-s001.zip › medicina-4365366-supplementary.pdf]
